# Disruption of *rcnB* modulates colistin susceptibility in *Acinetobacter baumannii* AB5075

**DOI:** 10.1080/21505594.2026.2697100

**Published:** 2026-07-14

**Authors:** Yuying Zhang, Jiabao Xing, Hang Zhang, Jingchun Kong, Sue C. Nang, Meng Zhang, Yushan Pan, Yajun Zhai, Li Yuan, Jinxin Zhao, Hua Wu

**Affiliations:** a Department of Pharmacology and Toxicology, College of Veterinary Medicine, Henan Agricultural University, Zhengzhou, China; b School of Public Health, Fudan University, Key Laboratory of Public Health Safety, Ministry of Education, Shanghai, China; c Department of Clinical Laboratory, Key Laboratory of Clinical Laboratory Diagnosis and Translational Research of Zhejiang Province, The First Affiliated Hospital of Wenzhou Medical University, Wenzhou, China; d Department of Microbiology, Monash Biomedicine Discovery Institute, Monash University, Clayton, Australia; e Microbiology Laboratory, Henan Centers for Disease Prevention and Control, Zhengzhou, Henan, China

**Keywords:** *Acinetobacter baumannii*, AB5075, *rcnB* gene, CRISPR-Cas9, colistin, gene editing

## Abstract

*Acinetobacter baumannii* AB5075 is a clinically relevant multidrug-resistant (MDR) isolate that poses a major therapeutic challenge. Although colistin has been reinstated as a last-resort antibiotic against MDR Gram-negative infections, the rapid emergence of colistin resistance threatens its clinical utility. Here, we employed a CRISPR-Cas9-based genome editing system to generate an *A. baumannii* AB5075 Δ*rcnB* mutant and uncovered a previously underappreciated role of *rcnB* in modulating colistin susceptibility. Loss of *rcnB* markedly potentiated colistin-mediated killing through multiple associated changes, including compromised membrane integrity, impaired oxidative stress defenses, and reduced efflux pump activity. Transcriptomic profiling further revealed that *rcnB* deletion reshaped global stress-response networks, including suppression of fatty acid biosynthesis and reactive oxygen species (ROS)-detoxifying pathways, alongside altered metal ion and sulfur metabolism during colistin exposure. Collectively, our findings suggest that *rcnB* may contribute to colistin susceptibility of colistin resistance and provide mechanistic insights that may inform the development of targeted strategies to enhance colistin efficacy against MDR *A. baumannii*.

## Graphical abstract

**Figure:**
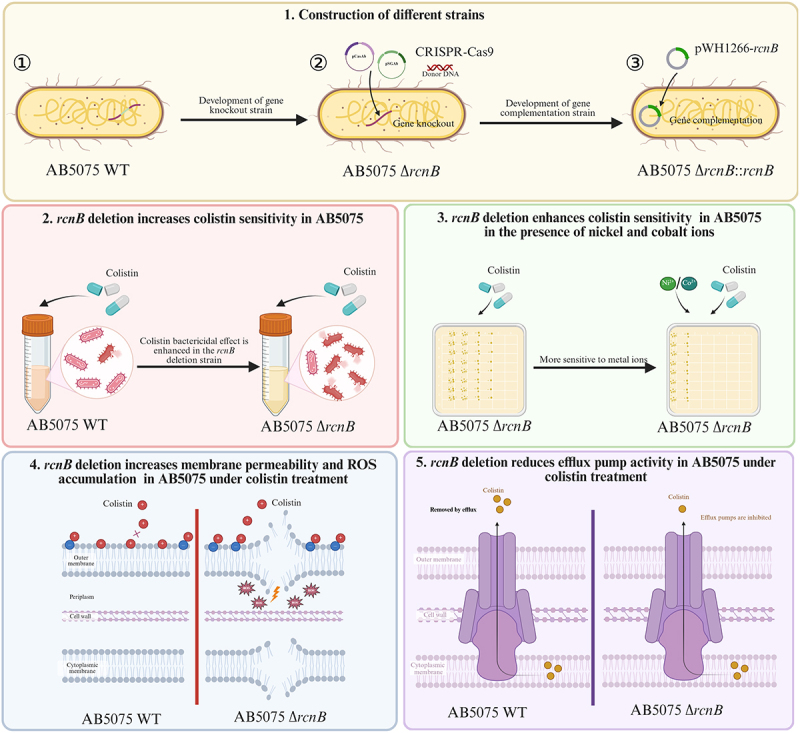

## Introduction

*Acinetobacter baumannii* has emerged as a leading cause of life-threatening hospital-acquired infections, including ventilator-associated pneumonia, bloodstream infections, and wound infection [1,2]. Its remarkable ability to accumulate antimicrobial resistance determinants has rendered many conventional therapies ineffective [3], positioning carbapenem-resistant *A. baumannii* (CRAB) among the World Health Organization’s highest-priority pathogens for urgent therapeutic development [4]. Historically, *A. baumannii* has predominantly been studied using a limited number of laboratory strains, which fail to accurately represent the resistance and virulence of multidrug-resistant (MDR) clinical isolates [5]. In contrast, the MDR clinical isolate AB5075 exhibits robust antimicrobial resistance and high virulence *in vivo*, providing a clinically relevant model for dissecting the molecular basis of *A. baumannii* pathogenesis and resistance [6].

Colistin, a critical last-line therapeutic option for MDR Gram-negative infections, exerts its antibacterial effect by binding to lipopolysaccharides (LPS) on the outer membrane (OM), disrupting membrane integrity and causing bacterial cell death [7–10]. However, the increasing prevalence of colistin resistance in *A. baumannii*—including both chromosomally encoded and plasmid-borne mechanisms – threatens its clinical utility [11–13]. Although current knowledge primarily centers on lipid A modifications mediated by the PmrAB-PmrC axis and other lipid remodeling pathways, these mechanisms do not fully explain the heterogeneity of colistin resistance observed among clinical isolates [5,14]. This gap suggests that additional intrinsic regulatory elements likely modulate the colistin stress response. Defining such regulators is essential to understanding how *A. baumannii* adapts to colistin-mediated membrane damage and to identifying molecular targets that can restore or potentiate colistin efficacy. Recent work has identified bacteriophage-mediated dissemination of phosphoethanolamine transferase genes, revealing an additional layer of mobile genetic control over colistin resistance [15]. These findings highlight the evolutionary plasticity of *A. baumannii* and the contribution of horizontal gene transfer to membrane remodeling strategies that mitigate polymyxin stress. Importantly, the AB5075 strain used in this study lacks the resistance-associated bacteriophage, thereby offering a genetically ideal system in which intrinsic, chromosomally encoded determinants of colistin susceptibility can be interrogated in isolation from phage-driven effects.

The *rcnB* gene, initially characterized in *Escherichia coli* as part of the RcnR-RcnB metal detoxification system involved in nickel and cobalt homeostasis, has remained largely unexplored in *A. baumannii* [16]. In *E. coli*, the Rcn system (RcnRAB) is involved in maintaining intracellular nickel and cobalt homeostasis, where RcnA functions as an efflux pump and RcnB is thought to modulate this process to help balance intracellular metal levels. Previous studies suggest that loss of *rcnB* may alter metal accumulation, supporting a role for RcnB in fine-tuning RcnA-mediated efflux [17,18]. Our preliminary work suggested a potential role of *rcnB* in modulating colistin susceptibility in AB5075 [19]; however, whether *rcnB* contributes to intrinsic colistin tolerance in *A. baumannii* and the molecular pathways through which it may do so remain unknown. Addressing this question is particularly compelling, as metal-linked signaling and redox-associated stress responses are increasingly recognized as critical determinants of antibiotic tolerance [20,21] yet have not been mechanistically connected to colistin susceptibility in *A. baumannii*.

CRISPR-Cas9 genome editing offers a powerful approach for functional genetic interrogation in bacteria [22–27]. While this system has been successfully applied in *A. baumannii*, its implementation in clinically relevant strains such as AB5075 can still be challenging due to strain-specific biological features, including genomic plasticity, phase variation, and high intrinsic resistance, which may affect editing efficiency [6,28–31]. Addressing these challenges in future studies will facilitate more robust genetic manipulation and support mechanistic investigations in such isolates.

In this study, we employed the CRISPR-Cas9/RecAb system, in which Cas9-mediated DNA cleavage is coupled with RecAb-dependent homologous recombination [32], to generate an AB5075 Δ*rcnB* mutant and systematically investigate the contribution of *rcnB* to colistin susceptibility. Through integrated phenotypic assays and transcriptomic analysis, we show that *rcnB* plays a previously underappreciated role in colistin resistance in *A. baumannii*. Our findings suggest a potential link between *rcnB* and membrane homeostasis, oxidative stress adaptation, and efflux-mediated defense, providing insights into how intrinsic stress-response pathways may be leveraged to enhance colistin efficacy against MDR *A. baumannii*.

## Materials and methods

### Strains, plasmids, primers, and culture conditions

The primers used in this study were synthesized by Qingke and were listed in Table S1. The AB5075 strain used in this study was provided by the Department of Infectious Diseases, Sir Run Run Shaw Hospital, College of Medicine, Zhejiang University. The *E. coli* DH5α strain used for plasmid construction was purchased from Tolobio. Unless otherwise stated, *A. baumannii* AB5075 and *E. coli* DH5α were routinely cultured in LB medium at 37°C. When appropriate, antibiotics were added at the following final concentrations: ampicillin (100 µg/mL), hygromycin (250 µg/mL), and tetracycline (50 µg/mL). For induction of RecAb recombinase and Cas9 nuclease expression, 1 mM isopropyl β-D-1-thiogalactopyranoside (IPTG) was supplemented into the culture.

### Construction of AB5075 engineered strain using CRISPR-Cas9 dual-plasmid system

All primers used in this study are listed in Table S1. The pCasAb-apr (Addgene #121998) and pSGAb-spe (Addgene #122000) plasmids were obtained from Addgene. To construct the CRISPR-Cas9/RecAb-based genome editing system, a 20-bp spacer was introduced into the BsaI-digested pSGAb-spe backbone via Golden Gate Assembly (NEB, Cat E1602) using the primers detailed in Table S1. Subsequently, the spectinomycin resistance cassette of pSGAb-spe was replaced with a hygromycin B resistance gene, yielding the pSGAb-hyg-spacer plasmid.

For genetic manipulation in *A. baumannii* AB5075 wild-type (WT) and the Δ*rcnB* mutant, electrocompetent cells were prepared as follows. Cultures were grown to an OD_600_ of 0.5–0.7, chilled on ice for 20 min, and harvested by centrifugation at 4°C and 8,000 rpm for 5 min. The pellet was washed three times with ice-cold 10% glycerol and finally resuspended in the same buffer. Aliquots (100 μL per 2-mL tube) were used for immediate electroporation or snap-frozen in liquid nitrogen and stored at −80°C. Competent cell preparation and electroporation of AB5075 harboring pCasAb-apr were performed following the protocol described by Quanjiang Ji et al [33].

For genome editing, the pSGAb-hyg-spacer plasmid and repair template (≤10 μL mixture) were electroporated into AB5075 cells pre-carrying pCasAb-apr. Transformants were selected on LB agar supplemented with the appropriate antibiotics and screened by PCR to identify positive clones (Table S1). Dual-plasmid curing was then conducted as previously described by Ji et al [33]. Successful loss of both plasmids and correct genomic edits were confirmed by PCR using the primers *rcnB*-s-F and *rcnB*-s-R [32].

### Gene complementation

The *rcnB* gene was PCR-amplified from the AB5075 genomic DNA using KOD high-fidelity polymerase (TOYOBO, Shanghai, China). The purified PCR product was inserted into the EcoRI-digested pWH1266 plasmid (Bosai Biotech) via In-Fusion seamless cloning, generating the pWH1266-*rcnB* construct [34]. The resulting plasmid was introduced into electrocompetent *A. baumannii* AB5075 Δ*rcnB* cells by electroporation. Following recovery, the cells were plated on LB agar supplemented with tetracycline (50 µg/mL) and incubated overnight at 37°C. Putative transformants were screened by PCR to confirm the presence of pWH1266-*rcnB*.

### Bacterial growth characteristics and antimicrobial susceptibility testing

#### Antimicrobial susceptibility assay

The *in vitro* antibacterial activity of colistin was evaluated using the broth microdilution method, following the guidelines of the Clinical and Laboratory Standards Institute (CLSI). Briefly, cation-adjusted Mueller – Hinton broth (CAMHB) was dispensed into 96-well microtiter plates, and a twofold serial dilution series of colistin was prepared. An equal volume of standardized bacterial suspension was added to each well. The plates were sealed and incubated at 37°C for 18 h, and the minimum inhibitory concentration (MIC) was subsequently determined [35].

#### Time-kill kinetics assays

The time-kill curve assay was conducted using three strains: AB5075 WT, AB5075 Δ*rcnB*, and AB5075 Δ*rcnB::rcnB* (hereafter: complemented strain). Overnight cultures of bacteria were diluted (1:100) in CAMHB and grown to an OD_600_ of 0.5. The cultures were then distributed into the following treatment groups: CAMHB alone, CAMHB +2 µg/mL colistin, CAMHB +6 µg/mL colistin, CAMHB +25 µM Ni^2+^, CAMHB +25 µM Co^2+^, CAMHB +2 µg/mL colistin +25 µM Ni^2+^, and CAMHB +2 µg/mL colistin +25 µM Co^2+^. The cultures were incubated at 37°C, and samples were collected at 0, 1, 2, 4, and 24 hours, serially diluted, plated on LB agar, and viable counts were determined.

### Sensitivity to disinfectants

Overnight bacterial cultures were subcultured in LB medium and grown at 37°C with shaking to an OD_600_ of approximately 0.15. Aliquots of 990 μL culture were then mixed with 10 μL of the corresponding disinfectants (benzethonium chloride (BZT), 0.01%; chlorhexidine gluconate (CHG), 0.008%; all purchased from Macklin). The mixtures were incubated at room temperature for 30 min. Subsequently, cultures were serially diluted and plated for colony-forming unit (CFU) enumeration [36].

### Fluorescence-based cellular response assays

The AB5075 WT, AB5075 Δ*rcnB*, and AB5075 Δ*rcnB::rcnB* strains were cultured at 37°C to the logarithmic phase, harvested by centrifugation (8,000 rpm, 5 min), washed 2–3 times with PBS, and resuspended to an OD_600_ of 0.5. Colistin was added to the cultures at final concentrations of 2, 4, or 6 µg/mL, followed by incubation at 37°C for 2 h. After treatment, the following fluorescence-based assays were performed:

**OM Permeability**: 1-N-phenylnaphthylamine (NPN) was added and samples were incubated at 37°C for 30 min. Fluorescence was recorded at 350/420 nm (Ex/Em).

**Cell Membrane Integrity**: Propidium iodide (PI) was added and incubated at 37°C for 30 min. Fluorescence was measured at 535/615 nm (Ex/Em).

**Reactive oxygen species (ROS)**: DCFH-DA (10 µM) was added to detect intracellular ROS. After 30 min at 37°C, fluorescence was measured at 488/525 nm (Ex/Em).

**Efflux Pump Activity**: Ethidium bromide (EtBr, 5 µM) was added to evaluate efflux function. Fluorescence was detected at 530/600 nm (Ex/Em).

### Transcriptomic analysis

To investigate the transcriptional responses of genetically engineered *A. baumannii* AB5075 strains to colistin, transcriptomic analysis was performed on the AB5075 WT, AB5075 Δ*rcnB*, and AB5075 ΔrcnB::rcnB*rcnB:rcnB* strains. Bacterial cultures were treated with colistin (4 µg/mL) or left untreated and incubated at 37°C for 4 h. Cells were harvested by centrifugation (4°C, 10,000 rpm, 2 min), rapidly frozen in liquid nitrogen, and stored at −80°C until RNA extraction.

Total RNA was extracted, and RNA integrity and purity were assessed using an Agilent 2100 Bioanalyzer and a NanoDrop spectrophotometer. Samples with RNA integrity number (RIN) ≥7.0 and OD_260/280_ ratios of 1.8–2.2 were used for library preparation. Ribosomal RNA was removed, and strand-specific cDNA libraries were prepared following standard protocols. Libraries were sequenced using an Illumina platform to generate 150 bp paired-end reads.

Raw reads were quality-filtered using Trimmomatic (v0.39) to remove adapter sequences and low-quality bases. High-quality clean reads were aligned to the *A. baumannii* AB5075 reference genome (GenBank accession: CP113078.1) using HISAT2 (v2.2.1). Gene expression levels were quantified with StringTie (v2.1.4) and normalized as fragments per kilobase of transcript per million mapped reads (FPKM). Differentially expressed genes (DEGs) were identified using DESeq2 (v1.36.0), with thresholds of |log_2_ fold change| ≥1 and a false discovery rate (FDR) <0.05. Functional classification and enrichment analyses of DEGs were carried out based on Gene Ontology (GO), Kyoto Encyclopedia of Genes and Genomes (KEGG), and Clusters of Orthologous Groups (COG) databases. Enrichment analysis was performed using a hypergeometric test, and *p*-values were adjusted for multiple comparisons using the Benjamini–Hochberg method. Pathways with an adjusted *p*-value < 0.05 were considered significantly enriched. Data visualization included heatmaps, Venn diagrams, and volcano plots generated using TBtools (v1.098) and R packages “pheatmap” and “ggplot2.” Principal component analysis (PCA) and hierarchical clustering were performed to assess transcriptional variation among strains and treatment conditions.

### RT-qPCR

To validate the expression of the deleted and complemented *rcnB* gene, Real-Time quantitative PCR (RT-qPCR) was performed on the AB5075 WT, AB5075 Δ*rcnB*, and AB5075 ΔrcnB::rcnB*rcnB:rcnB* strains. Total RNA was extracted using a commercial RNA extraction kit, and RNA concentration and purity were determined spectrophotometrically. cDNA was synthesized from 1 µg of total RNA using a reverse transcription kit following the manufacturer’s instructions. RT-qPCR was carried out using gene-specific primers, with the 16S rRNA gene serving as the internal reference for normalization. All reactions were performed in triplicate to ensure data reproducibility. Relative gene expression levels were calculated using the 2^−ΔΔCt method. Primer sequences used for RT-qPCR are listed in Table S1.

## Results

### Construction and verification of AB5075 ΔrcnB and complemented strain AB5075 ΔrcnB::rcnB

**Figure 1.**
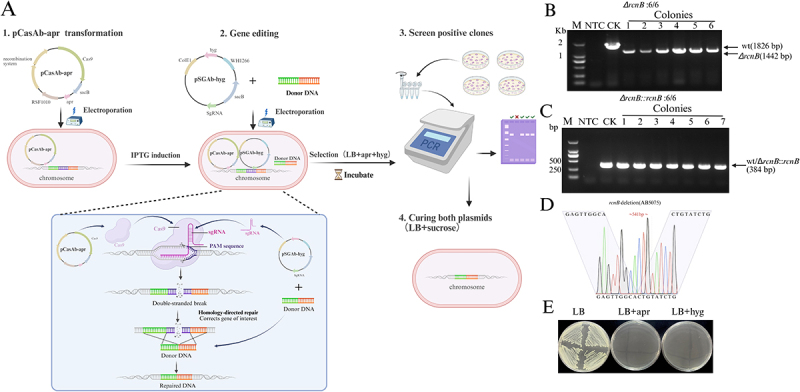
Genome editing in *A. baumannii* AB5075 using the pCasAb-pSGAb dual-plasmid system. A. Schematic representation of the CRISPR-Cas9/RecAb-based dual-plasmid genome editing strategy. B. *rcnB* gene knockout in AB5075 strain. C. *rcnB* gene complementation in AB5075 Δ*rcnB* strain. D. Sanger sequencing confirmation of successful deletion of a 541 bp fragment within the *rcnB* gene. E. Selection procedure for the elimination of pCasab-apr and pSgab-hyg plasmids using LB agar plates supplemented with 5% (w/v) sucrose. (Created with bioRender.com). M, DNA marker; NTC, no template control; WT, wild-type strain.

Our previous studies suggested that the *rcnB* gene may contribute to colistin susceptibility in *A. baumannii* [19]. To further elucidate its role, an *rcnB* deletion mutant (AB5075 Δ*rcnB*) and the corresponding complemented strain (AB5075 ΔrcnB::rcnB*rcnB:rcnB*) were constructed in the AB5075 background using a CRISPR-Cas9/RecAb-based dual-plasmid editing system, in which Cas9-mediated DNA cleavage is coupled with RecAb-dependent homologous recombination [32] (Figure 1(A)). Successful gene deletion and complementation were confirmed by PCR and Sanger sequencing (Figure 1(A–D)). The editing plasmids were subsequently cured prior to downstream experiments (Figure 1(E)). These validated strains were then used for subsequent phenotypic and mechanistic analyses.

### The deletion of the rcnB gene enhances the activity of colistin

The MICs of colistin for the AB5075 WT, AB5075 Δ*rcnB*, and AB5075 Δ*rcnB::rcnB* strains were determined to be 2 µg/mL, 0.5 µg/mL, and 2 µg/mL, respectively. Loss of *rcnB* markedly reduced the MIC, whereas complementation restored colistin susceptibility to the WT level, indicating that *rcnB* contributes to colistin resistance in *A. baumannii* [19].

**Figure 2.**
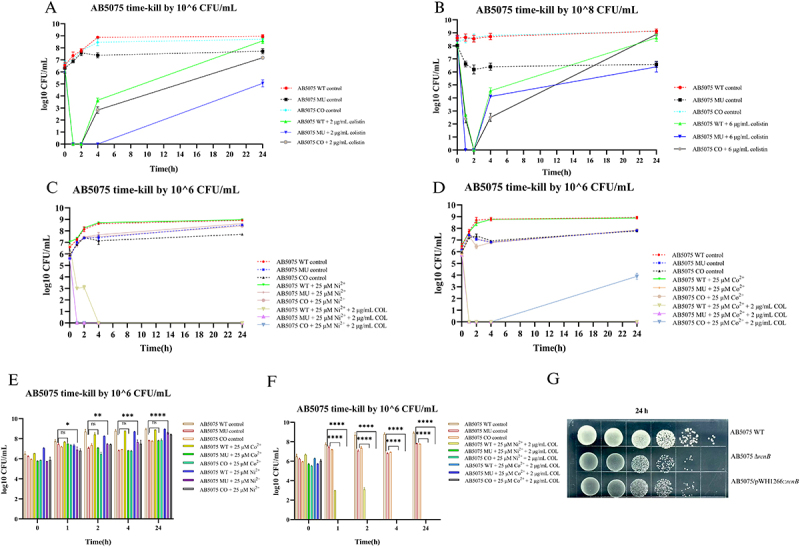
Impact of *rcnB* on colistin susceptibility in *A. baumannii*. A. Time-kill curves of AB5075 WT (wild-type), AB5075 MU (Δ*rcnB*), and AB5075 CO (ΔrcnB::rcnB*rcnB:rcnB*) strains following treatment with 2 µg/mL colistin. B. Time-kill curves of the indicated strains following treatment with 6 µg/mL colistin. C. Time-kill curves of AB5075 WT, AB5075 MU, and AB5075 CO strains following co-treatment with 2 µg/mL colistin and 25 µM Ni^2+^. D. Time-kill curves of the indicated strains following co-treatment with 2 µg/mL colistin and 25 µM Co^2+^. E. Time-kill curves of AB5075 WT, AB5075 MU, and AB5075 CO strains following treatment with 25 µM Ni^2+^ and 25 µM Co^2+^. F. Time-kill curves of AB5075 WT, AB5075 MU, and AB5075 CO strains following combined treatment with colistin and Ni^2+^/Co^2+^. G. Growth of AB5075 WT, AB5075 MU, and AB5075 CO strains on LB agar plates after 24 h of incubation at 37°C. All data are presented as mean ± standard deviation (SD) from three independent biological replicates. Statistical significance was determined using Student’s *t*-test at the indicated time points. ***p < 0.001, ****p < 0.0001.

Consistent with the MIC findings, time-kill assays further demonstrated the increased colistin sensitivity of the Δ*rcnB* mutant (Figure 2(A–B)). At 2 µg/mL colistin, AB5075 Δ*rcnB* displayed a rapid decline in viability within the first 0–4 h, with delayed regrowth observed only after 4 h. In contrast, both the WT and complemented strains exhibited earlier recovery by 4 h. Exposure to 6 µg/mL colistin resulted in an even more pronounced effect: AB5075 Δ*rcnB* showed an immediate > 3-log_10_ reduction in CFU within 1 h, and the bacterial load remained significantly lower than that of the WT and complemented strains over 24 h (Figure 2(A–B), 2(G)). Collectively, these results demonstrate that deletion of *rcnB* markedly compromises the ability of AB5075 to withstand colistin treatment.

Given the mechanistic similarity between disinfectants and colistin, as well as the clinical relevance of *A. baumannii* [37] we next assessed whether *rcnB* deletion alters susceptibility to commonly used disinfectants. Consistent with the enhanced sensitivity observed under colistin treatment, the Δ*rcnB* mutant exhibited markedly reduced survival compared with the wild-type strain upon disinfectant exposure. In contrast, complementation of *rcnB* partially restored resistance to levels comparable to the wild type (Figure S1). Notably, these findings indicate that *rcnB* contributes more broadly to stress tolerance in *A. baumannii*, particularly under conditions that disrupt membrane integrity and promote oxidative stress.

Given that *rcnB* has previously been implicated in nickel and cobalt homeostasis in *E. coli*^16–18^, we hypothesized that metal ion stress may similarly modulate colistin susceptibility in *A. baumannii*. Time-kill assays were therefore performed in the presence of Ni^2+^ or Co^2+^ (Figure 2(C–F)). Under nickel supplementation, both AB5075 Δ*rcnB* and AB5075 ΔrcnB::rcnB*rcnB:rcnB* exhibited dramatically enhanced sensitivity to colistin, with complete eradication of bacterial cells observed within 1 h and no regrowth at 24 h (Figure 2(F)). Notably, cobalt supplementation produced an even stronger bactericidal effect, eliminating all three strains including the wild type within 1 h, with no detectable regrowth during the subsequent 24 h (Figure 2(F)). These findings suggest that Ni^2+^ and Co^2+^ distinctly potentiate colistin activity, with cobalt exerting a broader inhibitory effect on bacterial survival and resistance pathways than nickel.

### rcnB deletion enhances membrane permeability, increases oxidative damage, and inhibits efflux pump activity in AB5075

**Figure 3.**
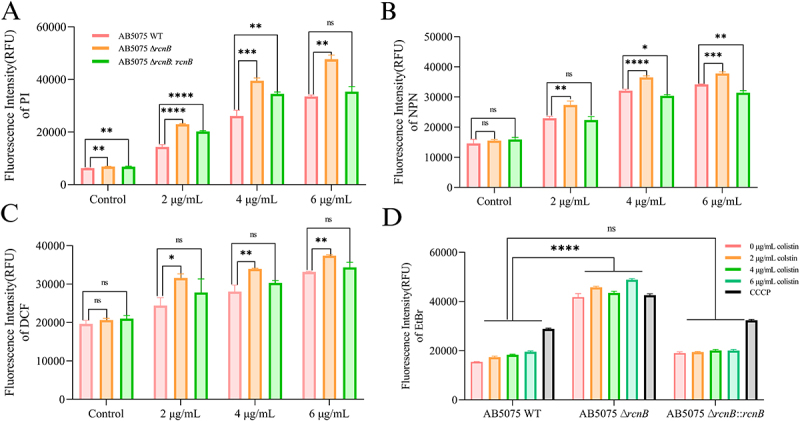
Membrane permeability, ROS production and efflux pump inhibition in AB5075 WT, AB5075 Δ*rcnB*, and AB5075 Δ*rcnB::rcnB* following colistin treatment. A. Inner membrane permeability of AB5075 WT, AB5075 Δ*rcnB*, and AB5075 ΔrcnB::rcnB*rcnB:rcnB* strains assessed using PI following treatment with colistin at concentrations of 2, 4, and 6 µg/mL. B. OM permeability of the indicated strains evaluated using NPN following colistin treatment at 2, 4, and 6 µg/mL. C. ROS production in AB5075 WT, AB5075 Δ*rcnB*, and AB5075 ΔrcnB::rcnB*rcnB:rcnB* strains measured using DCFH-DA following treatment with colistin at the indicated concentrations. D. Efflux pump activity assessed by EtBr accumulation in the indicated strains following colistin treatment at 2, 4, and 6 µg/mL. CCCP was deployed as the positive control. Data are presented as mean ± SD from three technical replicates, and representative of at least two independent experiments. Statistical significance was determined using one-way ANOVA followed by Tukey’ s multiple-comparison test.

Bacterial resistance to colistin is primarily driven by modifications in LPS and reduced affinity between colistin and OM components [38,39]. Preliminary antibiotic susceptibility testing and time-kill assays indicated that deletion of the *rcnB* gene enhances the bactericidal effect of colistin, likely by promoting membrane permeability. Building upon this observation, we hypothesized that *rcnB* deletion enhances colistin-mediated membrane permeability, thus amplifying its bactericidal activity. Using NPN and PI fluorescence assays, we found that colistin treatment (2, 4, or 6 µg/mL for 2 h) led to significantly higher fluorescence signals in AB5075 Δ*rcnB* than in the WT or complemented strains (Figure 3(A–B)), indicating increased OM permeability and compromised membrane integrity in the absence of *rcnB*.

In particularly, colistin may promote the accumulation of ROS within *A. baumannii* cells, contributing to its bactericidal effect, as ROS excessive production induces oxidative stress, which triggers lipid peroxidation and damages crucial biomolecules, including proteins and DNA, ultimately leading to bacterial cell death [40]. To explore this, we assessed intracellular ROS levels in AB5075 WT, Δ*rcnB* mutant, and complemented strains following exposure to varying concentrations of colistin for 2 hours. Consistent with transcriptomic analysis, the AB5075 Δ*rcnB* mutant displayed increased ROS accumulation, as evidenced by a marked enhancement in fluorescence intensity compared to both the WT and complemented strains (Figure 3(C)), suggesting impaired ROS detoxification in the Δ*rcnB* mutant.

We further evaluated efflux pump function by monitoring EtBr accumulation. Loss of *rcnB* resulted in significantly higher EtBr fluorescence following colistin exposure (Figure 3(D)), indicative of impaired efflux activity [41]. Although colistin partially inhibited efflux in the WT strain, the effect was substantially more pronounced in the Δ*rcnB* mutant. At 6 µg/mL, however, EtBr accumulation was comparable across all strains, suggesting that high-dose colistin overrides efflux-associated differences.

Taken together, these findings demonstrate that *rcnB* deletion sensitizes AB5075 to colistin, likely driven by enhanced membrane permeability, increased ROS accumulation, and reduced efflux pump activity. To further elucidate the transcriptional mechanisms underlying these phenotypes, we next performed comparative transcriptomic analysis under colistin exposure.

### Transcriptomic features of AB5075 WT, AB5075 ΔrcnB, and AB5075 δrcnb:rcnb after colistin treatment

**Figure 4.**
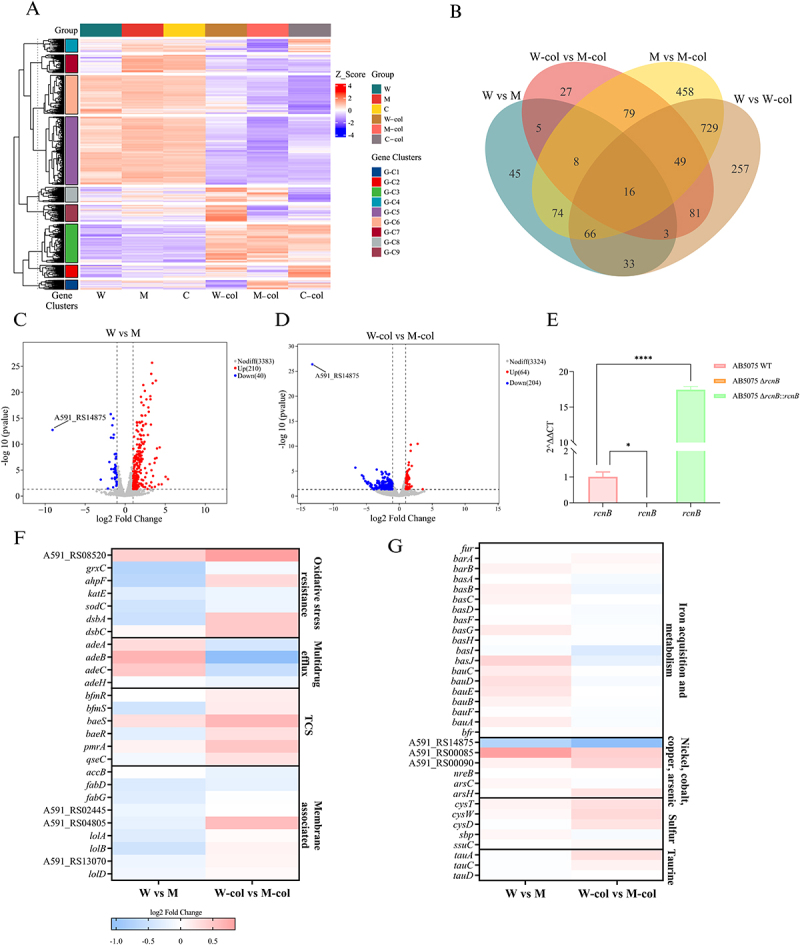
Transcriptomic profiling of *A. baumannii* AB5075 WT, AB5075 Δ*rcnB*, and AB5075 ΔrcnB::rcnB*rcnB:rcnB* under colistin selective pressure. A. Heatmap showing DEGs among AB5075 WT, AB5075 Δ*rcnB*, and AB5075 ΔrcnB::rcnB*rcnB:rcnB* strains following colistin treatment (4 µg/mL), revealing a global downregulation of gene expression. B. Venn diagram showing the differences in gene expression between AB5075 WT and AB5075 Δ*rcnB* under various treatment conditions. C. Volcano plot depicting DEGs between AB5075 WT and AB5075 Δ*rcnB* without colistin treatment. D. Volcano plot of DEGs between AB5075 WT and AB5075 Δ*rcnB* after colistin treatment (4 µg/mL). E. Gene expression levels of *rcnB* in AB5075 WT, AB5075 Δ*rcnB*, and AB5075 ΔrcnB::rcnB*rcnB:rcnB* strains. F. Expression patterns of DEGs involved in oxidative stress response, multidrug efflux, two-component systems, and membrane regulation. G. Expression of genes associated with metal and other metabolite homeostasis. Transcriptomic analysis was performed using three independent biological replicates per condition. Differential gene expression analysis was conducted using DESeq2, with genes exhibiting |log_2_ Fold change| ≥1 and FDR < 0.05 considered statistically significant. W represents AB5075 WT; M represents AB5075 Δ*rcnB*; C represents AB5075 ΔrcnB::rcnB*rcnB:rcnB*; W-col refers to AB5075 WT treated with 4 µg/mL colistin; M-col refers to AB5075 Δ*rcnB* treated with 4 µg/mL colistin; C-col refers to AB5075 ΔrcnB::rcnB*rcnB:rcnB* treated with 4 µg/mL colistin.

To characterize the global transcriptional impact of *rcnB* deletion on colistin responsiveness, RNA-seq was conducted on AB5075 WT, AB5075 Δ*rcnB*, and AB5075 ΔrcnB::rcnB*rcnB:rcnB* strains with or without colistin exposure (4 µg/mL, 4 h). RT-qPCR analysis supported the RNA-seq results, showing stable *rcnB* transcription in both the WT and complemented strains, whereas no expression was detected in the AB5075 Δ*rcnB* mutant (Figure 4(E)). Hierarchical clustering revealed distinct transcriptional profiles among the six groups, with colistin treatment causing a marked shift in gene expression across all strains, predominantly characterized by gene downregulation (Figure 4(A)). Comparative analysis identified 49 shared DEGs across all colistin-treated groups, while strain-specific alterations were also evident. Specifically, 45 DEGs distinguished AB5075 WT from AB5075 Δ*rcnB* mutant under basal conditions, and 27 DEGs remained unique to this comparison after colistin treatment (Figure 4(B)). Volcano plot analysis further highlighted the extent of transcriptional remodeling (Figure 4(C–D)). Under untreated conditions, Δ*rcnB* displayed 210 upregulated and 40 downregulated genes compared with WT. Upon colistin exposure, the expression pattern was reversed, with 204 genes downregulated and only 64 upregulated, indicating a dramatic transcriptional repression upon loss of *rcnB*. The complete differential expression datasets comparing AB5075 WT and AB5075 Δ*rcnB* strains under untreated and colistin-treated conditions are provided in Supplementary Tables S2-S3, respectively. Notably, *rcnB* (A591_RS14875) itself displayed significant differential expression across conditions, consistent with its regulatory involvement (Figure 4(B–C)). Collectively, these findings indicate that mRNA profiles of all strains were significantly altered upon colistin exposure, with the Δ*rcnB* strain showing a substantial increase in downregulated genes compared to the WT strain (Figure 4(A); Figure 4(C-D)).

To dissect functional consequences, DEGs were categorized into stress response, membrane homeostasis, metal ion metabolism, and sulfur utilization pathways (Figure 4(F–G); Figure S3). Compared with colistin-treated WT, the Δ*rcnB* mutant exhibited marked downregulation of genes associated with ROS detoxification, including *katE*, *sodC*, and *grxC* [7,42], indicating impaired antioxidant defense (Figure S2). In contrast, protein-folding machineries such as *dsbA* and *dsbC* family genes were upregulated, reflecting enhanced protein damage and misfolding stress. Colistin also affected membrane-associated functions, with reduced expression of the AdeABC RND efflux pump and altered regulation of two-component systems (*baeSR*, *bfmSR*) and membrane assembly genes (*lolABCD*, *mlaD*), consistent with compromised membrane integrity and stress signaling in Δ*rcnB* [43,44]. Comparative transcriptomic analyses between untreated and colistin-treated conditions within the WT and Δ*rcnB* backgrounds further demonstrated that colistin exposure was associated with broad alterations in the expression of genes involved in oxidative stress response, membrane-associated pathways, and metal ion metabolism (Figure S3). Compared with the WT background, these transcriptional alterations were more pronounced in the Δ*rcnB* mutant under colistin treatment, particularly among genes involved in iron acquisition and sulfur metabolism.

Beyond membrane and oxidative stress pathways, loss of *rcnB* was associated with transcriptional changes in genes related to metal ion and sulfur metabolism (Figure 4(G)). The Δ*rcnB* mutant exhibited reduced expression of several genes involved in iron acquisition, including siderophore biosynthesis and transport (*bar-bas-bau* cluster) and bacterioferritin gene *bfr* [21], suggesting altered regulation of iron metabolism. These findings were consistently supported by both RT-qPCR and transcriptomic analyses (Figure S2). Under colistin treatment, the Δ*rcnB* mutant exhibited more pronounced transcriptional changes in genes associated with iron acquisition and metabolism, as well as sulfur metabolism, compared with the untreated condition (Figure S3). Interestingly, several siderophore-associated genes, including *barA*, *bauA*, and *bauD*, displayed distinct expression trends under untreated and colistin-treated conditions, suggesting that disruption of *rcnB* may differentially influence iron acquisition-associated responses depending on antibiotic stress conditions. In addition, deletion of *rcnB* led to compensatory upregulation of the two remaining RcnB-family paralogs (A591_RS00085 and A591_RS00090). Furthermore, genes involved in sulfate transport and assimilation (*cysWD*) and sulfur-containing amino acid metabolism, including taurine catabolism (*tauACD*), were significantly upregulated, indicating metabolic rewiring under colistin pressure.

Collectively, transcriptomic profiling indicates that *rcnB* deletion is associated with coordinated transcriptional adjustments involving oxidative stress response, membrane-related pathways, efflux systems, metal ion regulation, and sulfur metabolism. These coordinated transcriptional changes mechanistically align with the heightened colistin sensitivity observed in the Δ*rcnB* mutant.

## Discussion

The rapid evolution of MDR Gram-negative pathogens has reinforced the clinical value of colistin as a critical last-line therapy [45,46]. Yet, the global dissemination of plasmid-borne *mcr* genes has severely compromised its efficacy, highlighting an urgent need to identify endogenous regulatory mechanisms that modulate colistin susceptibility [47]. Consistent with this concern, our previous studies have demonstrated that the deletion of the *rcnB* gene in AB5075 significantly enhances its susceptibility to colistin [19] although the precise molecular mechanisms underlying this phenomenon remain poorly understood. It is worth noting that *rcnB* has been characterized in *E. coli* as a key regulator of metal ion homeostasis; however, its functional relevance in *A. baumannii* and potential link to colistin resistance remain unexplored [17,18]. In this study, we successfully generated an AB5075 Δ*rcnB* mutant using a CRISPR-Cas9/RecAb-based genome editing system. Importantly, we further demonstrate that loss of *rcnB* markedly increases colistin susceptibility in *A. baumannii*, identifying *rcnB* as a previously unrecognized intrinsic resistance determinant and providing insights into the cellular processes underlying colistin response.

While CRISPR-Cas-mediated editing has been applied to several bacterial species, genetic manipulation of *A. baumannii* AB5075 remains technically challenging due to its robust intrinsic resistance and complex genomic plasticity [6,48–51]. In this study, we successfully generated the Δ*rcnB* mutant in AB5075 using a CRISPR-Cas9-based genome editing system. This work provides mechanistic insights into colistin resistance and demonstrates the practical application of CRISPR-Cas9-based editing for functional genomics in AB5075 and potentially other clinically relevant *A. baumannii* lineages.

Mechanistically, we demonstrate that loss of *rcnB* profoundly enhances colistin-mediated killing through a multifactorial process. First, Δ*rcnB* cells exhibited pronounced membrane vulnerability, as evidenced by increased NPN and PI fluorescence, consistent with disrupted lipid homeostasis. Transcriptomic profiling further revealed downregulation of fatty acid biosynthesis genes (e.g. *accB*, *fabDGI*), linking *rcnB* deletion to impaired membrane biogenesis. Second, Δ*rcnB* mutants accumulated significantly higher levels of intracellular ROS under colistin exposure, accompanied by the suppression of ROS detoxification genes (*katE*, *sodC*, *grxC*) [52–54], indicating a weakened antioxidant defense that magnifies oxidative damage. In parallel, Δ*rcnB* displayed diminished efflux capacity, supported by both EtBr efflux assays and downregulation of RND efflux system genes. Notably, increased intracellular EtBr accumulation was observed even in the absence of colistin, indicating a basal defect in efflux activity rather than a solely drug-induced phenomenon. This impaired transport function may promote intracellular retention of colistin and other toxic compounds, further sensitizing the mutant strain. These alterations were accompanied by activation of stress-responsive two-component systems (*baeSR*, *bfmSR*), suggesting compensatory signaling triggered by membrane perturbation and oxidative imbalance [55]. In addition to colistin, deletion of *rcnB* also increased susceptibility to commonly used disinfectants, suggesting a broader role in stress tolerance. Notably, the time-kill assays revealed a biphasic pattern characterized by rapid bacterial killing followed by apparent regrowth at later time points [19]. We observed that the Δ*rcnB* mutant exhibited a degree of growth impairment even in the absence of colistin, which may partially influence the interpretation of killing dynamics. The regrowth observed after an initial decline to very low CFU levels may not necessarily indicate the emergence of stable resistant mutants. Instead, it could reflect recovery from a small surviving population that was initially below the detection limit or phenotypic adaptation under prolonged incubation. Therefore, while Δ*rcnB* clearly shows increased susceptibility to colistin, the late-stage regrowth should be interpreted with caution, and further validation would be required to distinguish between genetic resistance and transient tolerance. These observations do not allow us to distinguish between stable genetic resistance and transient phenotypic adaptation. Collectively, our data suggest that *rcnB* deletion sensitize*s A. baumannii* to colistin and commonly used disinfectants by concurrently compromising membrane integrity, antioxidant defense, and efflux-mediated protection. Importantly, the transcriptomic analyses in this study were performed following exposure to colistin at 4 μg/mL for 4 h, a condition selected to capture early stress-associated transcriptional remodeling rather than terminal bactericidal events. Under these experimentally optimized conditions established through preliminary laboratory assays, bacterial counts decreased during treatment; however, complete bacterial eradication was not observed within the 4 h exposure period. Previous transcriptomic and metabolomic studies investigating colistin responses have similarly employed comparable exposure windows and demonstrated substantial alterations in oxidative stress, membrane remodeling, and adaptive stress-response pathways during this stage [56–58]. Therefore, the transcriptional profiles observed here likely reflect population-level adaptive responses to colistin-induced stress rather than transcriptional signatures derived exclusively from a small residual survivor population. Nonetheless, because antibiotic exposure may generate physiologically heterogeneous bacterial subpopulations, the RNA-seq results should be interpreted as representing an integrated stress-response landscape at the population level rather than a uniform cellular state.

In *E. coli*, the Rcn system (RcnRAB) is involved in maintaining intracellular nickel and cobalt homeostasis. RcnA functions as an efflux pump, while RcnB is thought to modulate this process to help balance intracellular metal levels. Previous studies suggest that loss of *rcnB* may alter metal accumulation, supporting a role for RcnB in fine-tuning RcnA-mediated efflux [16–18]. Our work indicates that *A. baumannii* has adapted this conserved metal-sensing system to perform broader physiological functions beyond Ni^2+^/Co^2+^ homeostasis [17]. Transcriptomic profiling of the Δ*rcnB* mutant revealed marked perturbations in iron uptake and sulfur-linked redox metabolism-two pathways tightly connected to oxidative stress control, lipid A remodeling and envelope resilience [19]. Notably, similar transcriptional activation of sulfur metabolism has been observed in *A. baumannii* under desiccation stress, a condition associated with membrane damage and ROS accumulation, where cysteine-mediated redox buffering contributes to oxidative stress tolerance [59]. Because colistin lethality relies heavily on inducing oxidative damage and compromising membrane integrity, these disruptions likely weaken the bacterium’s ability to restore redox balance and maintain a stable envelope under antibiotic pressure. Notably, this model aligns with findings from experimental evolution studies, which similarly demonstrate that perturbations in membrane remodeling and redox homeostasis strongly shape *A. baumannii*’ s adaptive trajectories under polymyxin selection [60].

Taken together, these findings position RcnB in *A. baumannii* as a regulatory node that integrates metal-sensing signals with global stress-response pathways to support colistin tolerance. Disrupting *rcnB* appears to destabilize this interconnected network, resulting in heightened susceptibility to colistin-mediated killing. This expanded functional role not only distinguishes RcnB from its canonical homeostasis-oriented activity in *E. coli* but also highlights metal-ion-linked signaling as a previously unrecognized contributor to intrinsic colistin tolerance. As such, RcnB-associated pathways represent promising mechanistic targets for therapeutic exploitation.

Our study has noteworthy limitations. Although transcriptomic profiling provided mechanistic clues, the conclusions are primarily inferred from gene expression. Integrating lipidomics, metallomics, and real-time ROS tracing will be critical to validate causality [21,61]. Furthermore, while Ni^2+^ and Co^2+^ were observed to markedly potentiate colistin-mediated killing across all strains, the underlying mechanisms remain incompletely understood. Based on existing literature, Ni^2+^ and Co^2+^ effects may be attributed to metal-induced membrane destabilization and oxidative stress. Specifically, cobalt has been shown to disrupt bacterial membranes, leading to the loss of membrane integrity, leakage of intracellular contents, and induction of oxidative stress [62]. Similarly, nickel toxicity has been associated with lipid peroxidation (LPO) and the generation of ROS, which may further exacerbate colistin-induced damage [63]. These metal-induced stresses could act in parallel with colistin, enhancing its bactericidal activity. However, the precise connection between this mechanism and *rcnB* remains unclear, and further studies are required to validate these proposed mechanisms. In addition, the lack of *in vivo* infection models limits our ability to directly assess the contribution of *rcnB* to bacterial virulence and fitness under host conditions. Whether *rcnB* exerts similar regulatory functions in other high-priority pathogens such as *Klebsiella pneumoniae* or *Pseudomonas aeruginosa* remains unknown. Future work should assess the conservation of *rcnB*-mediated stress adaptation across species and evaluate whether pharmacological inhibition of *rcnB* or its downstream pathways can potentiate colistin activity in MDR isolates.

In summary, this work identifies *rcnB* as a previously unrecognized intrinsic regulator of colistin susceptibility in *A. baumannii* and provides mechanistic insight into how its deletion potentiates colistin bactericidal activity. By coupling CRISPR-based genome editing with phenotypic and transcriptomic analyses, we establish *rcnB* as a promising therapeutic target whose inhibition could restore or enhance colistin efficacy against MDR *A. baumannii*, offering a new avenue for intervention in the era of rising colistin resistance.

## Supplementary Material

reviewer comments.docx

Table S2 Differentially expressed genes between AB5075 WT and AB5075 ΔrcnB without colistin treatment.xlsx

Fig S1.png

Table S3 Differentially expressed genes between AB5075 WT and AB5075 ΔrcnB after colistin treatment.xlsx

Fig S2.png

Fig S3.png

Table S1 Primers used in the st.xlsx

Legends for All Supplementary Figures and Tables.docx

## Data Availability

Transcriptome data have been deposited in the CNGB (China National GeneBank) database with the accession number [CNP0008585] and are available at the CNGB website (https://db.cngb.org/data_resources/?query=CNP0008585) [64]. All other data supporting the findings of this study have been deposited in the figshare repository (DOI: 10.6084/m9.figshare.30877154) and are accessible at https://doi.org/10.6084/m9.figshare.30877154 [65].
