# Supplementary figures and images for: Disruption of rcnB modulates colistin susceptibility in Acinetobacter baumannii AB5075
Source: Virulence. 2026 Jul 14;17(1):2697100. doi: 10.1080/21505594.2026.2697100 (PMC13371490; doi:10.1080/21505594.2026.2697100)

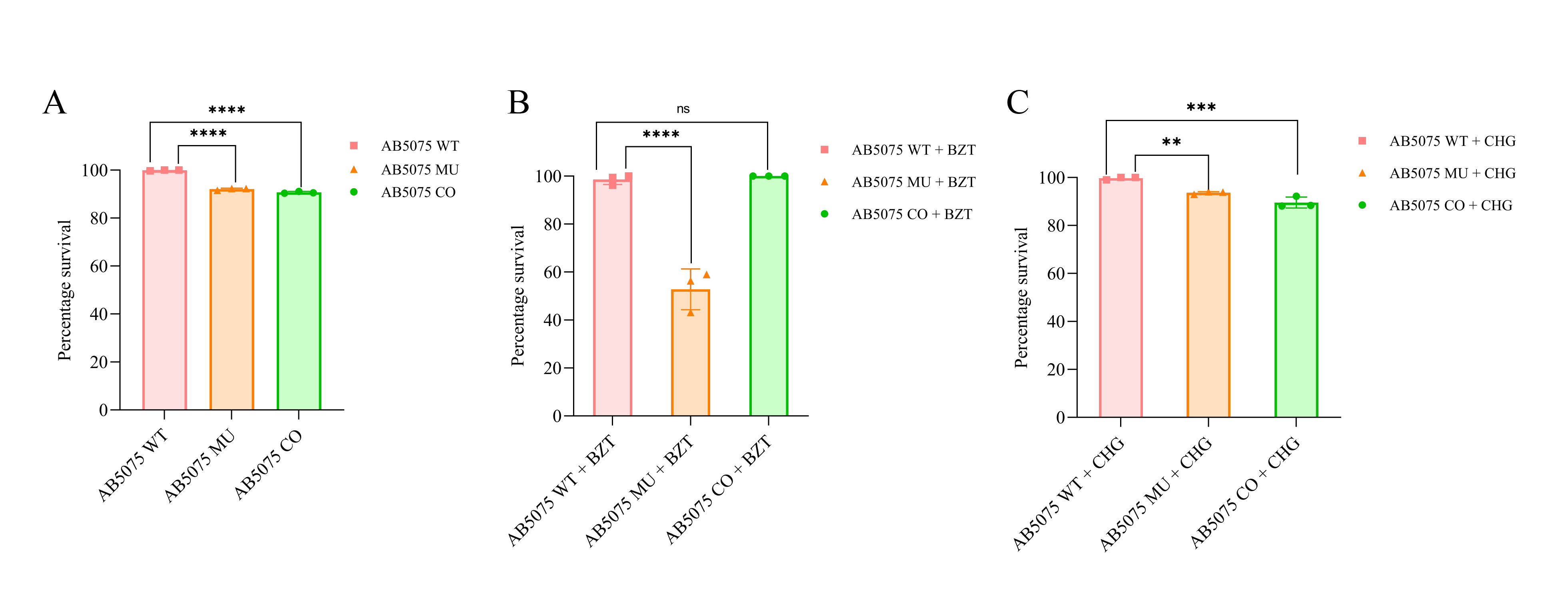

Supplement: Fig S1.png [file KVIR_A_2697100_SM8516.png]

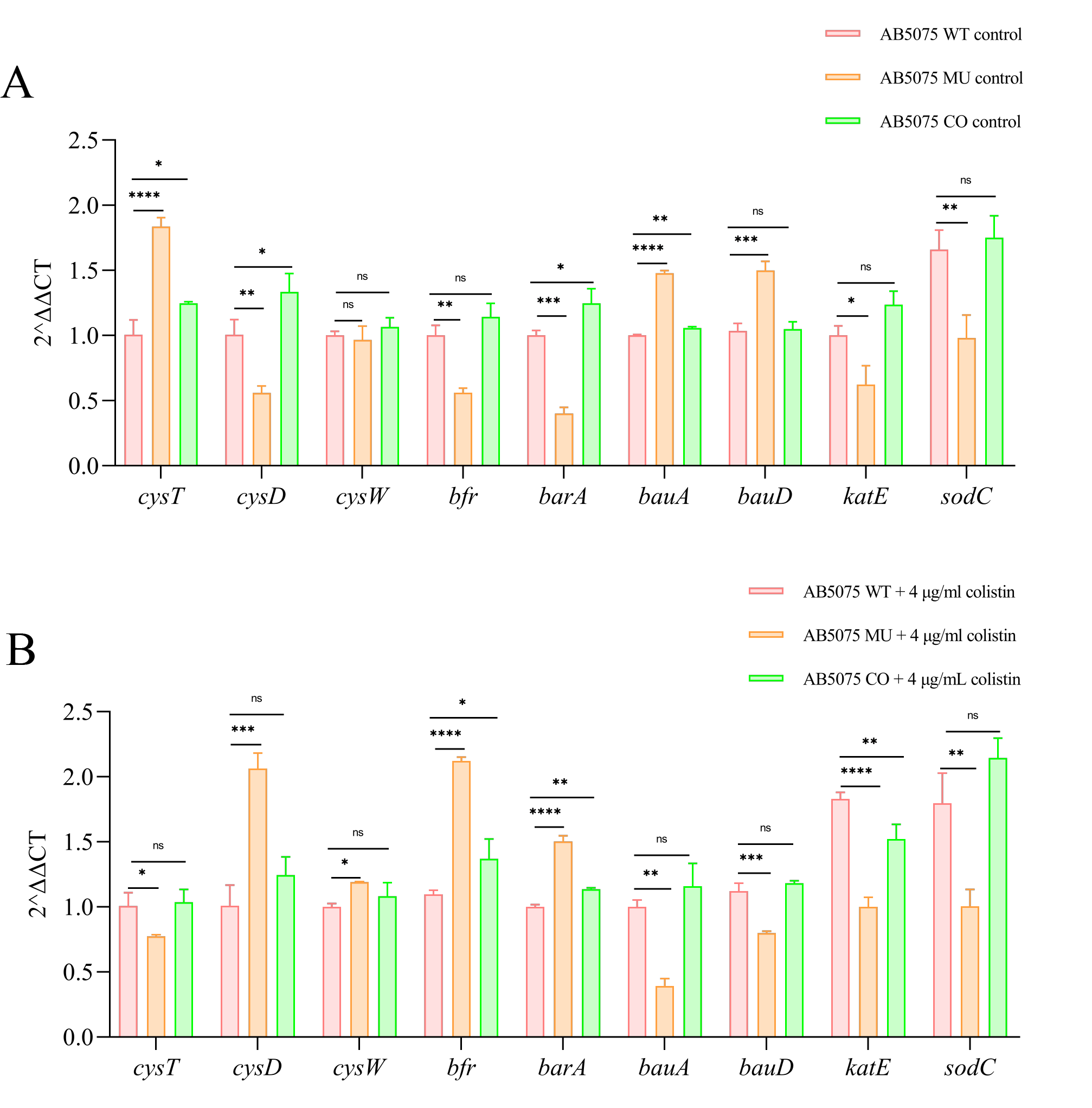

Supplement: Fig S2.png [file KVIR_A_2697100_SM8514.png]

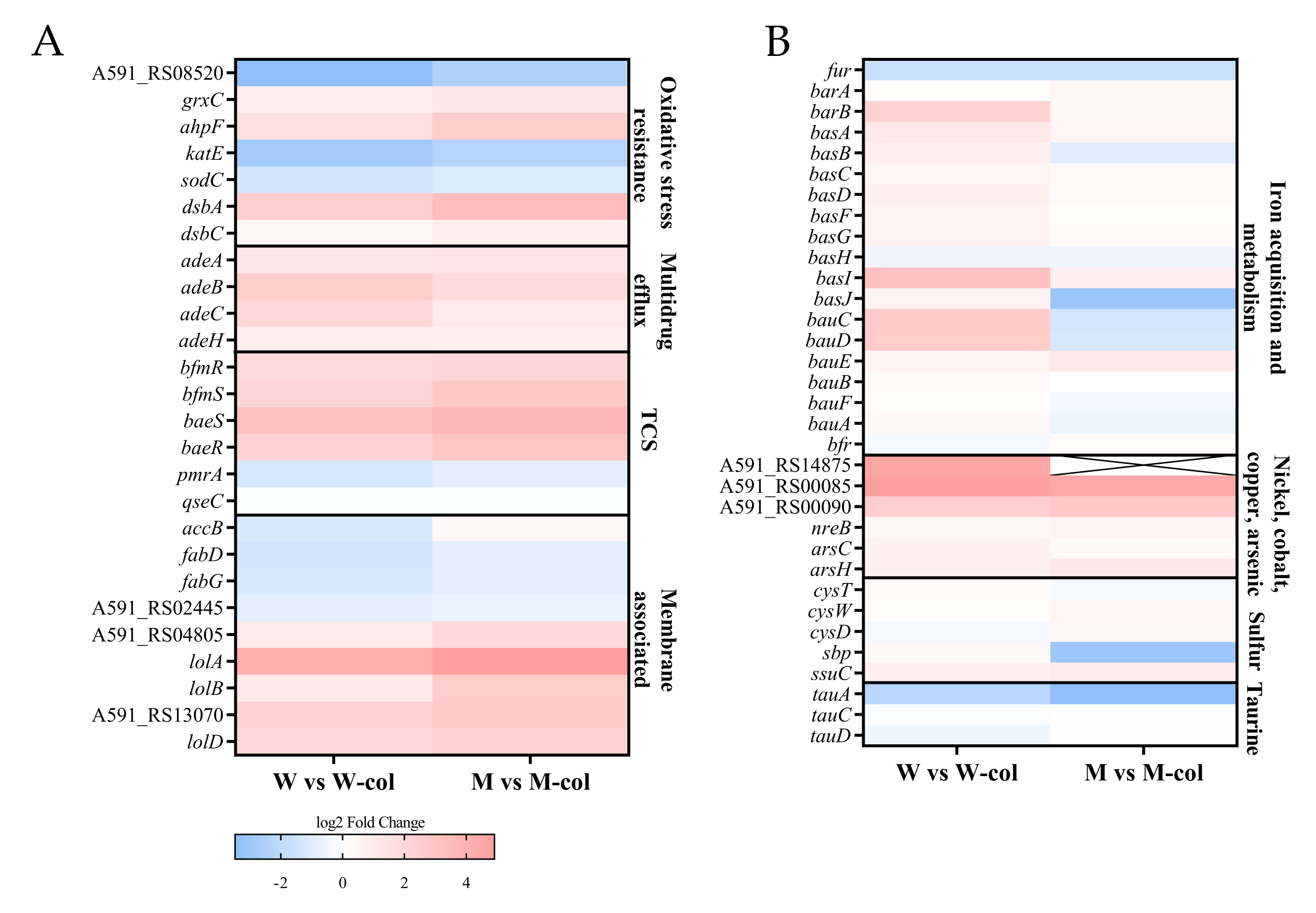

Supplement: Fig S3.png [file KVIR_A_2697100_SM8513.png]
