# Supplementary material for: Disruption of rcnB modulates colistin susceptibility in Acinetobacter baumannii AB5075
Source: Virulence. 2026 Jul 14;17(1):2697100. doi: 10.1080/21505594.2026.2697100 (PMC13371490; doi:10.1080/21505594.2026.2697100)
Supplement: Legends for All Supplementary Figures and Tables.docx [file KVIR_A_2697100_SM8511.docx]

Figure S1. Survival of AB5075 WT (wild-type), AB5075 MU (Δ*rcnB*), and AB5075 CO (Δ*rcnB*::*rcnB*) under disinfectant treatment. (A) Untreated control. (B) Cells treated with benzethonium chloride (BZT) (0.01%). (C) Cells treated with chlorhexidine gluconate (CHG) (0.008%). Percentage survival was calculated relative to AB5075 WT (wild-type). Statistical significance was determined using Student’ s t-test at the indicated time points. ***p < 0.001, ****p < 0.0001.

Figure S2. RT-qPCR analysis of genes associated with iron acquisition, sulfur metabolism, and oxidative stress in AB5075 WT, Δ*rcnB*, and complemented strains under untreated (A) and colistin-treated (B) conditions.

Figure S3. Differential expression profiles of genes associated with oxidative stress response, multidrug efflux systems, two-component regulatory systems, and membrane homeostasis (A), as well as metal ion and metabolite homeostasis (B).

Table S1. Primers used in the study.

Table S2. Differentially expressed genes between AB5075 WT and AB5075 Δ*rcnB* without colistin treatment.

Table S3. Differentially expressed genes between AB5075 WT and AB5075 Δ*rcnB* after colistin treatment.
