# Supplementary material for: Disruption of rcnB modulates colistin susceptibility in Acinetobacter baumannii AB5075
Source: Virulence. 2026 Jul 14;17(1):2697100. doi: 10.1080/21505594.2026.2697100 (PMC13371490; doi:10.1080/21505594.2026.2697100)
Supplement: reviewer comments.docx [file KVIR_A_2697100_SM8518.docx]

Dear reviewers,

Thank you for the opportunity to revise our manuscript. We sincerely appreciate the reviewers’ careful evaluation and constructive comments, which have helped us to improve both the clarity and scientific rigor of our work. In response to the reviewers’ comments, we have thoroughly revised the manuscript. The major changes are summarized below:

**Abstract and Terminology**

- Revised full names and abbreviations throughout the Abstract and main text for consistency.
- Moderated the description of *rcnB* to avoid overstatement of its role as a metal regulator.
- Introduction
- Standardized terminology and abbreviation usage.
- Expanded the background on phage and membrane-associated mechanisms related to colistin resistance.
- Moved relevant discussion of *rcnB* from *E. coli* into the Introduction to better establish biological context.
- Revised statements related to CRISPR systems to avoid overemphasis and ensure consistency with existing literature.
- Moderated descriptions of *rcnB* function to avoid unsupported claims.

**Methods**

- Clarified that genome editing was performed using a CRISPR-Cas9/RecAb-based system.
- Added detailed methodological descriptions for disinfectant susceptibility assays.
- Included statistical methods for enrichment analysis (hypergeometric test with Benjamini-Hochberg correction).

**Results**

- Standardized terminology and corrected figure labeling errors.
- Revised CRISPR system description for consistency.
- Added results related to disinfectant susceptibility.
- Included additional transcriptomic analyses (WT vs WT + COL and MU vs MU *+* COL) to directly demonstrate transcriptional responses to colistin Figure R3 (Figure S3 in ms).
- Clarified that Ni^2+^/Co^2+^ enhance colistin activity across all strains, indicating a largely *rcnB-*independent effect.

**Discussion**

- Standardized terminology and refined CRISPR-related descriptions.
- Moderated statements regarding genetic editing in AB5075 to avoid overemphasis.
- Expanded discussion on disinfectant susceptibility and stress adaptation.
- Clarified the interpretation of regrowth observed in time-kill assays.
- Incorporated discussion of desiccation-related stress responses.
- Integrated *rcnB*’ s putative role in metal-associated stress responses with transcriptomic findings, while avoiding overinterpretation.
- Clarified that Ni^2+^ and Co^2+^ likely potentiate colistin activity through parallel, *rcnB-*independent mechanisms.
- Added discussion of study limitations, including the absence of *in vivo* infection models and the need for further mechanistic investigation.

We believe that these revisions have addressed all reviewer concerns and have significantly improved the clarity, balance, and rigor of the manuscript.

Enclosed please find the revised manuscript and our detailed point-by-point responses to the reviewers’ comments. We hope that the revised version is now suitable for publication and look forward to your consideration.

Thank you for your time and for handling our manuscript.

Yours sincerely,

Hua Wu, on behalf of all authors

**General comment from reviewer #1:**

Comments to the Author

The manuscript has been implemented to a sufficient level of rigor and completeness to warrant publication.

**Response:** We sincerely thank the reviewer for the positive evaluation of our manuscript. We are encouraged that the reviewer considers the study to have reached a sufficient level of rigor and completeness for publication.

**General comment from reviewer #2:**

Comments to the Author

The revision adequately addresses my critiques regarding several overstatements and the background on RcnB function, which has been improved; however, other criticisms were not sufficiently addressed. While text revisions were made, the part of the transcriptome experiment involving 4 hours of colistin treatment is still not able to be interpreted because this is presumably reflecting a small viable subpopulation, the nature of which (colistin-resistant mutants or not) remains undescribed.

**Response:** We thank the reviewer for this important comment and agree that interpretation of transcriptomic responses following colistin exposure requires careful consideration.

In this study, the RNA-seq experiments were performed following exposure to colistin at 4 µg/mL for 4 h. This condition was selected based on preliminary experimental evaluation and internal discussions within our research team^1-3^. Under these conditions, bacterial counts declined during treatment; however, bacterial eradication was not observed within the 4h exposure period.

The selected exposure window was intended to capture stress-associated transcriptional remodeling during colistin challenge. Previous transcriptomic and metabolomic studies investigating bacterial responses to colistin have similarly employed comparable exposure durations and reported alterations in oxidative stress, membrane remodeling, and adaptive stress-response pathways during this stage^4^. Consistent with these observations, our RNA-seq analyses also revealed broad transcriptional alterations following colistin exposure.

At the same time, we acknowledge that bacterial populations exposed to antibiotic stress may exhibit physiological heterogeneity, which could contribute to the integrated transcriptional signatures observed at the population level. We have therefore further moderated the interpretation of the RNA-seq findings and clarified this limitation in the revised Discussion section.

| **Original** | **Revised** |
| --- | --- |
| We observed that the Δ*rcnB* mutant exhibited a degree of growth impairment even in the absence of colistin, which may partially influence the interpretation of killing dynamics. The regrowth observed after an initial decline to very low CFU levels may not necessarily indicate the emergence of stable resistant mutants. Instead, it could reflect recovery from a small surviving population that was initially below the detection limit or phenotypic adaptation under prolonged incubation. Therefore, while Δ*rcnB* clearly shows increased susceptibility to colistin, the late-stage regrowth should be interpreted with caution, and further validation would be required to distinguish between genetic resistance and transient tolerance. These observations do not allow us to distinguish between stable genetic resistance and transient phenotypic adaptation. Collectively, our data suggest that *rcnB* deletion sensitizes *A. baumannii* to colistin and commonly used disinfectants by concurrently compromising membrane integrity, antioxidant defense, and efflux-mediated protection. | We observed that the Δ*rcnB* mutant exhibited a degree of growth impairment even in the absence of colistin, which may partially influence the interpretation of killing dynamics. The regrowth observed after an initial decline to very low CFU levels may not necessarily indicate the emergence of stable resistant mutants. Instead, it could reflect recovery from a small surviving population that was initially below the detection limit or phenotypic adaptation under prolonged incubation. Therefore, while Δ*rcnB* clearly shows increased susceptibility to colistin, the late-stage regrowth should be interpreted with caution, and further validation would be required to distinguish between genetic resistance and transient tolerance. These observations do not allow us to distinguish between stable genetic resistance and transient phenotypic adaptation. Collectively, our data suggest that *rcnB* deletion sensitizes *A. baumannii* to colistin and commonly used disinfectants by concurrently compromising membrane integrity, antioxidant defense, and efflux-mediated protection. Importantly, the transcriptomic analyses in this study were performed following exposure to colistin at 4 μg/mL for 4 h, a condition selected to capture early stress-associated transcriptional remodeling rather than terminal bactericidal events. Under these experimentally optimized conditions established through preliminary laboratory assays, bacterial counts decreased during treatment; however, complete bacterial eradication was not observed within the 4 h exposure period. Previous transcriptomic and metabolomic studies investigating colistin responses have similarly employed comparable exposure windows and demonstrated substantial alterations in oxidative stress, membrane remodeling, and adaptive stress-response pathways during this stage^56-58^. Therefore, the transcriptional profiles observed here likely reflect population-level adaptive responses to colistin-induced stress rather than transcriptional signatures derived exclusively from a small residual survivor population. Nonetheless, because antibiotic exposure may generate physiologically heterogeneous bacterial subpopulations, the RNA-seq results should be interpreted as representing an integrated stress-response landscape at the population level rather than a uniform cellular state. |

In addition:

-Fig. S3 is referenced in line 317 but the comparisons that the heatmap show are not actually described in the text.

**Response:** We thank the reviewer for this helpful comment. We have revised the Results section to include a brief description of the comparisons shown in Figure S3 (WT vs WT-col and Δ*rcnB* vs Δ*rcnB*-col) and the associated transcriptional changes under colistin treatment.

| **Original** | **Revised** |
| --- | --- |
| Colistin also affected membrane-associated functions, with reduced expression of the AdeABC RND efflux pump and altered regulation of two-component systems *(baeSR*, *bfmSR*) and membrane assembly genes (*lolABCD,* *mlaD*), consistent with compromised membrane integrity and stress signaling in Δ*rcnB*^43, 44^. | Colistin also affected membrane-associated functions, with reduced expression of the AdeABC RND efflux pump and altered regulation of two-component systems (*baeSR*, *bfmSR*) and membrane assembly genes (*lolABCD*, *mlaD*), consistent with compromised membrane integrity and stress signaling in Δ*rcnB*^43, 44^. Comparative transcriptomic analyses between untreated and colistin-treated conditions within the WT and Δ*rcnB* backgrounds further demonstrated that colistin exposure was associated with broad alterations in the expression of genes involved in oxidative stress response, membrane-associated pathways, and metal ion metabolism (Figure S3). Compared with the WT background, these transcriptional alterations were more pronounced in the Δ*rcnB* mutant under colistin treatment, particularly among genes involved in iron acquisition and sulfur metabolism. |

-Lines 315-316 state that transcriptional changes were more significant with colistin treatment, but it is not clear what this means. Fig. S2B shows that expression of *barA*, *bauA* and *bauD* expression changed in the mutant under colistin treatment, but the direction of the change was in the opposite direction from Fig. S2A (no colisitin treatment).

**Response:** We thank the Reviewer for this helpful comment.

(1) Clarification of transcriptional changes under colistin treatment

We agree that the phrase “more significant” was insufficiently precise and could lead to ambiguity. To clarify this point, we have revised the text to indicate that, under colistin treatment, the Δ*rcnB* mutant exhibited more pronounced transcriptional changes in genes associated with iron acquisition and metabolism, as well as sulfur metabolism, compared with the untreated condition. This revision more accurately reflects the transcriptional patterns observed in Figure S3.

(2) Clarification of condition-dependent expression patterns of iron acquisition-associated genes

We thank the Reviewer for this insightful observation. We agree that the expression patterns of *barA*, *bauA*, and *bauD* differed between the untreated and colistin-treated conditions. Specifically, in the absence of colistin, *barA* expression was reduced in the Δ*rcnB* mutant, whereas *bauA* and *bauD* showed increased expression. In contrast, following colistin exposure, *barA* expression increased, while *bauA* and *bauD* were reduced.

We believe these findings may reflect condition-dependent remodeling of iron acquisition-associated responses under antibiotic stress rather than a uniform transcriptional pattern. Previous studies have shown that iron homeostasis in *A. baumannii* is closely associated with oxidative stress adaptation and can undergo dynamic transcriptional regulation during antibiotic exposure^5^. Because iron acquisition may contribute both to stress adaptation and to ROS-associated toxicity, siderophore-associated genes may exhibit distinct regulatory patterns under different stress conditions.

In addition, given the established role of the Rcn system in nickel and cobalt homeostasis in *E. coli*, disruption of *rcnB* may indirectly influence broader metal-responsive regulatory pathways in *A. baumannii.* Such cross-regulation between Ni/Co-associated pathways and iron acquisition systems has been suggested in previous studies of bacterial metal homeostasis and oxidative stress adaptation^6, 7^. However, the precise mechanistic relationship between *rcnB*, Ni^2+^/Co^2+^-associated responses, and the observed transcriptional remodeling of iron acquisition genes remains unclear in the current study.

We agree that these observations are intriguing and warrant further investigation. However, detailed mechanistic dissection of this potential metal-associated regulatory interplay is beyond the scope of the present work and will be explored in future studies. We have revised the manuscript accordingly to clarify these condition-dependent transcriptional patterns and to avoid overinterpretation of the observed expression changes.

| **Original** | **Revised** |
| --- | --- |
| These findings were consistently supported by both RT-qPCR and transcriptomic analyses (Figure S2). Notably, under colistin treatment, these transcriptional changes were more significant, indicating an enhanced perturbation of metal ion–associated responses in the absence of *rcnB* (Figure S3). | These findings were consistently supported by both RT-qPCR and transcriptomic analyses (Figure S2). Under colistin treatment, the Δ*rcnB* mutant exhibited more pronounced transcriptional changes in genes associated with iron acquisition and metabolism, as well as sulfur metabolism, compared with the untreated condition (Figure S3). |
| The Δ*rcnB* mutant exhibited reduced expression of several genes involved in iron acquisition, including siderophore biosynthesis and transport (*bar*-*bas*-*bau* cluster) and bacterioferritin gene bfr^21^, suggesting altered regulation of iron metabolism.These findings were consistently supported by both RT-qPCR and transcriptomic analyses (Figure S2). | The Δ*rcnB* mutant exhibited reduced expression of several genes involved in iron acquisition, including siderophore biosynthesis and transport (*bar*-*bas*-*bau* cluster) and bacterioferritin gene bfr^21^, suggesting altered regulation of iron metabolism. These findings were consistently supported by both RT-qPCR and transcriptomic analyses (Figure S2). Under colistin treatment, the Δ*rcnB* mutant exhibited more pronounced transcriptional changes in genes associated with iron acquisition and metabolism, as well as sulfur metabolism, compared with the untreated condition (Figure S3). Interestingly, several siderophore-associated genes, including *barA*, *bauA*, and *bauD*, displayed distinct expression trends under untreated and colistin-treated conditions, suggesting that disruption of *rcnB* may differentially influence iron acquisition-associated responses depending on antibiotic stress conditions. |

-RNA-seq data and analysis are added as Table S2 but I did not have access to this table.

**Response:** We thank the reviewer for pointing this out. In the revised manuscript, the RNA-seq differential expression datasets have been provided as Supplementary Tables S2 and S3. Table S2 contains the DEGs between AB5075 WT and AB5075 Δ*rcnB* under untreated conditions, while Table S3 contains the DEGs identified after colistin treatment (4 µg/mL). These supplementary tables have now been included in the revised submission and are also cited in the main text.

| **Original** | **Revised** |
| --- | --- |
| Specifically, 45 DEGs distinguished AB5075 WT from AB5075 Δ*rcnB* mutnat under basal conditions, and 27 DEGs remained unique to this comparison after colistin treatment (Figure 4B). Volcano plot analysis further highlighted the extent of transcriptional remodeling (Figure 4C–D). Under untreated conditions, Δ*rcnB* displayed 210 upregulated and 40 downregulated genes compared with WT. Upon colistin exposure, the expression pattern was reversed, with 204 genes downregulated and only 64 upregulated, indicating a dramatic transcriptional repression upon loss of *rcnB*. | Specifically, 45 DEGs distinguished AB5075 WT from AB5075 Δ*rcnB* mutnat under basal conditions, and 27 DEGs remained unique to this comparison after colistin treatment (Figure 4B). Volcano plot analysis further highlighted the extent of transcriptional remodeling (Figure 4C–D). Under untreated conditions, Δ*rcnB* displayed 210 upregulated and 40 downregulated genes compared with WT. Upon colistin exposure, the expression pattern was reversed, with 204 genes downregulated and only 64 upregulated, indicating a dramatic transcriptional repression upon loss of *rcnB*. The complete differential expression datasets comparing AB5075 WT and AB5075 Δ*rcnB* strains under untreated and colistin-treated conditions are provided in Supplementary Tables S2-S3, respectively. |

***Reference***

1. Bian X, Li M, Liu X, Zhu Y, Li J, Bergen PJ, et al. Transcriptomic investigations of polymyxins and colistin/sulbactam combination against carbapenem-resistant Acinetobacter baumannii. *Computational and structural biotechnology journal*. 2024;**23**:2595-605.

2. Bian X, Zhu Y, Liu X, Li X, Li W, Zhao J, et al. Transcriptome-constrained genome-scale metabolic modeling reveals central carbon and amino acid metabolic reprogramming underlying colistin-sulbactam synergy in Acinetobacter baumannii. *Antimicrobial agents and chemotherapy*. 2026:e0184825.

3. Han ML, Liu X, Velkov T, Lin YW, Zhu Y, Li M, et al. Metabolic Analyses Revealed Time-Dependent Synergistic Killing by Colistin and Aztreonam Combination Against Multidrug-Resistant Acinetobacter baumannii. *Frontiers in microbiology*. 2018;**9**:2776.

4. Han ML, Zhu Y, Creek DJ, Lin YW, Gutu AD, Hertzog P, et al. Comparative Metabolomics and Transcriptomics Reveal Multiple Pathways Associated with Polymyxin Killing in Pseudomonas aeruginosa. *mSystems*. 2019;**4**.

5. Gadar K, de Dios R, Kadeřábková N, Prescott TAK, Mavridou DAI, McCarthy RR. Disrupting iron homeostasis can potentiate colistin activity and overcome colistin resistance mechanisms in Gram-Negative Bacteria. *Communications biology*. 2023;**6**:937.

6. Carballido Lopez A, Cunrath O, Forster A, Pérard J, Graulier G, Legendre R, et al. Non-specific interference of cobalt with siderophore-dependent iron uptake pathways. *Metallomics : integrated biometal science*. 2019;**11**:1937-51.

7. Koch D, Nies DH, Grass G. The RcnRA (YohLM) system of Escherichia coli: a connection between nickel, cobalt and iron homeostasis. *Biometals : an international journal on the role of metal ions in biology, biochemistry, and medicine*. 2007;**20**:759-71.
